# Health Literacy and Migrant Communities in Primary Health Care

**DOI:** 10.3389/fpubh.2021.798222

**Published:** 2022-01-24

**Authors:** Patrícia Medina, Ana Catarina Maia, Andreia Costa

**Affiliations:** ^1^Nursing Research, Innovation and Development Centre of Lisbon (CIDNUR), Nursing School of Lisbon (ESEL), Lisboa, Portugal; ^2^NOVA National School of Public Health, Public Health Research Centre, Universidade NOVA de Lisboa; Comprehensive Health Research Centre (CHRC), Lisboa, Portugal; ^3^Instituto de Saúde Ambiental (ISAMB), Faculdade de Medicina, Universidade de Lisboa, Lisboa, Portugal

**Keywords:** health literacy, health promotion, migrants, equity, primary health care

## Abstract

**Introduction:**

The promotion of health literacy of the population in a situation of migration, in the community, is a fundamental field of intervention in health promotion, for the reduction of inequalities in access to health care services. It is increasingly necessary to make health care services more equitable for migrant populations. The aim of the study was to characterize the level of health literacy of the population in a migrant situation, attending a primary health care unit in the Lisbon region, to identify priority areas for community intervention that will become the focus of intervention and contribute to the increase in the health literacy levels in this population.

**Methods:**

A cross-sectional study was carried out by applying the Health Literacy Survey (ILS-PT) to a sample of the population in a situation of migration, found by 27 participants.

**Results:**

The general health literacy index of the sample is inadequate (21.23 points). An analysis of the sub-indexes revealed that 75% of the participants had difficulties related to information about health care and 80% had difficulties in the field of health promotion.

**Conclusions:**

Problematic and inadequate levels of health literacy was significantly frequent among migrant population. So that enhancing health literacy among migrant is essential to reduce health inequalities to achieve better health outcomes and contribute to defense of human rights of this vulnerable population.

## Introduction

There are currently about 244 million migrants worldwide (1). A migrant is understood to be any person who moves or crosses an international border far from their place of habitual residence, regardless of their legal status, voluntary movement, causes of movement or length of stay (2).

In Portugal, data released indicate that, for the third consecutive year, there was an increase in the foreign population residing in Portugal, with an increase of 13, 9% compared to 2017 data, making a total of 480.3 thousand foreigners (3). The migrant population in Portugal tends to be concentrated in coastal regions, with the Metropolitan Area of Lisbon being the geographical area of the country with the highest percentage of foreign population (68.9%) (3).

The migratory process affects and is affected by the social determinants of health, as well as by the conditions in which the migratory process takes place, influencing the health situation, when leaving the countries of origin and when arriving in the countries of destination, influencing the process of integration in societies. Some studies developed in the European context and in Portugal shows the phenomenon “healthy migrant effect,” that is, the tendency of self-perception of a good health status upon arrival in the host countries. (4, 5). However, it is observed that throughout the time of stay in the host country, migrants tend to report a decline in their health status with reference to chronic diseases and disability (5, 6). In fact, studies conducted in Portugal on the health of migrants express the situation of greater vulnerability of certain groups to the development of chronic diseases and health problems with impact on the social determinants of health (5, 7). In fact, in some situations, the migration process can have a negative impact on the health status of the people involved, reinforcing their condition of vulnerability. This is the case of children and unaccompanied minors, women, elderly, people with disabilities, irregular migrants, refugees, asylum seekers, and migrants who have been subjected to human trafficking, so it is essential to understand their vulnerability and the impact it has on their health condition (8). Migrant health must be seen as a unified agenda, involving global health and universal health coverage, sustainable development goals and social determinants of health (9). In this sense, interventions must be designed to reduce inequities in health and increase the resilience of people in a situation of migration. Promoting health equity through universal health coverage with the broad participation of primary health care, ensures that improving the health of migrants is an essential part of achieving the Sustainable Development Goals addressed in the 2030 Agenda for Sustainable Development, adopted by all United Nations Member States in 2015. In this line of ideas, the design and implementation of interventions aimed at promoting the health and well-being of migrants, should have as an outcome the issue of equity on the agenda of health care providers, researchers in health sciences and in policies health care (10). Equity in health is of crucial importance for the defense of human rights in migrant populations, in view of the situation of increased vulnerability and their specific needs of this population at community and individual levels (10, 11). Aspects that for legal reasons, reduce access to health care and health information; and the environmental and integration issues of host countries, as well as the barriers related to communication, all have an impact on health equity (11).

Thus, promoting Health Literacy is a fundamental strategy for empowering citizens and promoting equity in access to health care, as described by the Portuguese National Health Plan: 2012–2016 revision and extension to 2020 (12), reducing its characteristic vulnerability associated with the migration process. In the Portuguese context, the Strategic Plan for Migration (13) (2015), structured by the Government of Portugal, highlights the universal access of migrant populations, regardless of their regularization status. On the other hand, the plan states that measures for the integration of migrants in Portugal should be structured and comprehensive for all health services, as universal access to health care by migrants and a better and more effective use of health services by migrant populations have been seen as “key indicators of social inclusion” and integration in the host country (14, 15). Initially, the concept of Health Literacy was defined by the World Health Organization as the set of cognitive and social skills that determine the motivation and ability of individuals to access, understand and use information to promote and maintain good health (16).

In the specific case of this study, the Integrated Conceptual Model of Health Literacy was used, in which health literacy is related to literacy and implies the knowledge, motivation and skills of the person to access, understand, evaluate and apply the health information in order to make judgments and make decisions on a daily basis relating to healthcare, disease prevention and health promotion in order to maintain or improve the quality of life over the course of life (17).

The health Literacy promotion process according to the present model requires four types of key competences: access, which refers to the ability to seek, find and obtain information about health; understand: referring to the ability to understand the health information that was obtained; evaluate: competence related to the ability to interpret, filter, judge and evaluate the health information that was accessed; apply: ability to communicate and use information that affect the decisions aimed at maintaining or improving health (17).

The population in a situation of migration is a naturally more vulnerable group, either because of the increased risk associated with the migration process itself and the conditions in which it occurs, or because of the situational conditions of integration in the host country, as access to health care and health information, that are determining factors for improving their health status (18).

Low health literacy and poor citizen autonomy toward health services are identified as “threats to equity and access to health care” by the National Health Plan 2012–2016 (19). This Plan proposes that literacy promotion actions be carried out, focused on measures to promote health and disease prevention, which can be developed and implemented by specialist community nurses (19).

The present study aims to describe the level of health literacy of a sample of migrant people who attend primary health care in a unit in the Lisbon region, to identify priority areas of community intervention initiated by specialist community nurses to increase literacy in health in these populations.

## Methods

### Study Design

This was a cross-sectional study conducted on a primary healthcare service, in Lisbon region, between 30th September and 16th October 2019. Ethical issues were validated by an independent commission before the data collection and was also obtained the approval from the executive director from the primary health structure.

### Study Participants

The population studied were persons that were in migration situation, and that resorted to a primary healthcare service in Lisbon region. A convenience sample of eligible participants was invited to participate in the study. Migrants were invited to participate through personal invitation when attending the primary healthcare service to receive nursing care. Were defined the following eligibility criteria: being over 18 years old; finding themselves in a situation of migration; have an oral proficiency of the Portuguese language; having accepted to participate in the study, after being explained by the researchers the contents of the study information sheet and after signing the written informed consent. Were included 27 participants in this study.

### Data Collection Procedure

Participants who met the eligibility criteria were invited to respond to the questionnaires during the time they remained at the health unit (before or after the appointments) and to return the completed questionnaires directly to the researchers. When distributing the data collection instrument, in the temporal episodes in which the participants were in the health unit, the researchers obtained and clarified the informed consent, addressing topics such as the objectives of the study, the rights of the participants and the methodology of data collection. The informed consent form was collected immediately before the participants filled out the questionnaires, and the anonymous coding of the questionnaires was carried out. All participants gave their informed consent to participate in this study and anonymity was assured.

The non-repetition of participation was guaranteed (since the questionnaires were anonymous), through the confirmation of a non-existence of a previous administrative contact record during the period of data collection For all the people who participated, they were offered help in explaining the questions formulated in the instrument, as well as help filling out the form.

### Measurements

#### Demographic Characteristics

Socio-demographic indicators assessed included age (date of birth), sex (female, male), birthplace (country of birth); educational level (up to the second cycle of primary education; up to the third cycle of primary education; high school; university education); employment status (working professionally; not working professionally); monthly income (≤500€; 501€−1,000€; 1,001€−1,500€; 1501€−2,000€).

#### Health Literacy Level

The validated version of Health Literacy Survey Portugal (ILS-PT) was used to measure migrant's health literacy. The questionnaire was developed by Professor Rita Espanha and her team (20). Permission to use the instrument was obtained from the authors. The ILS-PT consists of several modules. Module 1 (20), concerns the translation of the European Health Literacy Survey Questionnaire (HLS-EU-Q) (21)); it consists of 47 questions comprising four indexes. For its calculation, it is necessary to assign to each answer a certain classification, based on the difficulty of performing a certain health-related task (1- very difficult; 2- difficult; 3- easy; 4- very easy). The general health literacy index (comprising 47 items) GEN-HL (General Health Literacy Index) and three other indexes were calculated: the health care index (HC-HL—Health Care Literacy Index), with 16 items, the disease prevention index (DP-HL—Disease Prevention Literacy Index), with also 16 items and the health promotion index (HP-HL—Health Promotion Literacy Index), with 15 items (14). The indexes were calculated using a metric ranging from 0 to 50, where 0 represents the minimum of HL and 50 the maximum possible. The indexes were calculated according to the following formula: Index = (mean-1) × (50/3), where the mean refers to the mean of the answers of the items of each participant; value 1 is the minimum possible value of the mean and value 3 is the maximum value of the mean in the scale of difficulty in answering the questions asked (22).

In the case of the sample in question, the 27 questionnaires are valid, as they all have 100% response in the HLS items. The four indexes developed (GEN-HL, HP-HL, DP-HL and HP-HL) characterize the level of HL, according to the score obtained, as follows: from 0 to 25 points, corresponds to an inadequate level of HL; from 26 to 33 points, corresponds to a problematic HL level; from 34 to 42 points, corresponds to a HL sufficient level; and finally, from 43 to 50 points, corresponds to an excellent level of HL.

### Statistical Analysis

Analysis was conducted by using a software Version 25.0 SPSS 25 (The Statistical Package for the Social Sciences software) (Version 25.0., SPSS Inc., Chicago, IL). The sample of this study was characterized in terms of their sociodemographic characteristics, as well as by the rating of the different dimensions of Health Literacy, gotten according to the responses obtained through the ILS-PT, namely in healthcare, disease prevention and health promotion indexes. In this research, only descriptive statistics were used. Descriptive statistics and frequencies were performed to describe the variables under evaluation.

## Results

### Sociodemographic Characteristics

Table 1 shows the sociodemographic distribution of the participants. The average age of the participants was 31.6 years, with the predominant age group between 25 and 34 years of age (55.6%). The sample consisted of 85.2% women and 14.8% men. Regarding birthplace (and as birthplace, it is understood as the country of birth, as the ILS-PT refers), the most represented nationalities are the Brazil's people with 22.2%, Sao Tome's people with 18.5%, Angola's people with 18.5% and Guinea people with 14.8%. In terms of education level, degrees up to 2nd cycle (37%) and secondary education (33.3%) predominate, with higher education (18.5%) being in a minority. Regarding professional activity, 70.4% of the participants were professionally active and 29.6% were not active (which included participants with no professional activity, the unemployed, students or retired people). Finally, concerning to monthly income, about 59.3% of the participants have an income in their household which is equal to or < €1000 and about 29.6% of the participants have a monthly income equal to or less 500€.

**Table 1:** Sociodemographic characteristics.

**Variables**	***n* (%)**
Age (years)^*^	
18–24 25–34 35–54	3 (11.1%) 15 (55.6%) 9 (33.3%)
Sex	
Female Male	23 (85.2) 4 (14.8)
Birthplace	
Angola Guiné-Bissau Cabo Verde São Tomé e Príncipe Guiné-Conacri Brasil Venezuela Roménia Serra Leoa China	5 (18.5) 4 (14.8) 1 (3.7) 5 (18.5) 1 (3.7) 6 (22.2) 1 (3.7) 2 (7.4) 1 (3.7) 1 (3.7)
Education Level up to the second cycle of primary education up to the third cycle of primary education High school Higher education	10 (37) 3 (11.1) 9 (33.3) 5 (18.5)
Employment status	
Working professionally Not working professionally	19 (70.4) 8 (29.6)
Monthly income	
≤ 500€ 501€−1,000€ 1001€−1,500€ 1501€−2,000€	8 (29.6) 16 (59.3) 2 (7.4) 1 (3.7)

* *The mean age of the participants is 31.6 years*.

### Health Literacy Profile Assessment

Supplementary Table 2 shows the distribution of answers to the questions of module 1 by the matrix of the 12 sub-dimensions based on the HL Integrated Conceptual Model. Within the scope of the items related to health care (from question 1 to question 16), more than 50% of the participants refers that is “very difficult” or “difficult”: (a) find information about symptoms of illness that concern them (59.3%); (b) find information about treatments of illnesses that concern them (63%); (c) knowing what to do in case of a medical emergency (51.9%); (c) understand the leaflets that come with the medications (85.2%); (d) understand what to do in a medical emergency (59.3%); (e) judge the advantages and disadvantages of treatment options (88.9%); (f) assess the need for a second medical opinion (96.3%). In the same health care index, more than 50% of the participants considered it to be “easy” or “very easy”: (a) knowing where to get professional help when they are sick (88.9%); (b) understanding what their doctors tells them (55.6%), although the asymmetry is not significant against the opposite categories (44.4%); (c) understand the instructions from the doctor or pharmacist about taking a medication that was received (85.2%); (d) assess how the information given by your doctor applies to their clinical condition (55.6%); (e) assess whether information about diseases spread in the media is reliable (66.7%); (c) use doctor's information to decide about their illness (70.4%); (d) follow the instructions about the prescribed medication (92.6%); (e) call an ambulance in an emergency situation (55.6%); (f) follow the instructions of their doctor or pharmacist (92.6%).

When analyzing the items that refer to disease prevention (from question 17 to question 31), more than 50% of the participants reports that is “very difficult” or “difficult”: (a) finding information on how to manage mental health problems such as stress or depression (96.3%); (b) find information about vaccines and medical exams they should have to do (88.9%); (c) judge when to go to the doctor for a check-up or a general health exam (81.5%); (d) evaluate which vaccines they may need (92.6%); (e) evaluate which health screenings they have to do (88.9%).

In the same index on disease prevention, more than 50% of the participants consider that is “easy” or “very easy”: (a) finding information on how to manage unhealthy behaviors, such as smoking, lack of physical activity and excessive alcohol consumption (81.5 %); (b) find information on how to avoid or control situations such as overweight, high blood pressure and high cholesterol (88.9%); (c) understand health warnings about unhealthy behaviors such as smoking, low of physical activity and excessive alcohol consumption (92.6%); (d) understand the need of to be vaccinated (81.5%); (e) understand why they need to be seen by doctors in routine medical check-ups (63.0%); (e) assess how reliable health warnings are, such as smoking, lack of physical activity and excessive alcohol consumption (88.9%); (f) assess whether the information transmitted in the media about health risks is reliable (81.5%); (g) decide whether they should get the flu vaccine or not (59.3%); (h) deciding how to protect themselves from illnesses based on advice from family and friends (92.6%); (i) decide how to protect themselves from diseases based on information transmitted by the media (88.9%).

Finally, regarding health promotion, almost the total index of items were classified as “very difficult” or “difficult” (above 50%) by the participants, namely: (a) find information on activities that are beneficial to your mental well-being (92.6%); (b) find information about how their neighborhood can be more health-friendly (96.3%); (c) finding information about policy changes that can be addressed as health issues (96.3%); (d) find information about the efforts to promote their health at work (92.6%); (e) understand information on how to keep their mind healthy (92.6%); (f) assess how the place where they live affects their health and well-being (88.9%); (g) assess how the conditions of their home help them to stay healthy (66.7%); (h) assess which everyday behavior is related to their health (63.0%); (i) make decisions that improve their health (63.0%); (j) join a gym or a sport if they want to (81.5%); (k) change the living conditions that affect their health and well-being (92.6%); (l) participate in actions that improve health and well-being in their community (92.6%). In the same index the participants classified as “easy” or “very easy” the following items: (a) find information on healthy activities such as exercise, healthy food, and nutrition; (b) understanding health advice given by family and friends (96.3%); (c) understand information present in food packaging (59.3%); (d) understanding information in the media on to get healthier (100%).

### Levels of Health Literacy

Table 2 shows the values for the General Health Literacy Index (GEN-HL), the Health Care Literacy Index (HC-HL); the Disease Prevention Literacy Index (DP-HL) and the Health Promotion Literacy Index (HP-HL). The results show that the participants have on average, an inadequate level of health literacy in general ($\bar{x}$ = 21.2) (GEN-HL). Furthermore, 85.2% of the participants have an inadequate level health literacy; 11.1% have a problematic level and only 3.7% have a sufficient level. Regarding the Health Care Index (HC-HL), the participants maintain an average inadequate level of health care literacy ($\bar{x}$ = 25.3). Concerning this index, about 55.6% of the participants have an inadequate level, 37% have a problematic level and 7.4% have a sufficient level. About the Disease Prevention Index, it is shown that the participants have an average inadequate level ($\bar{x}$ = 25.02). About 66.7% of the participants have an inadequate level of health literacy in disease prevention; 25.9% have a problematic level and 7.4% have a sufficient level. Finally, in the Health Promotion Index (HP-HL) the participants have an average inadequate level of literacy related to disease prevention ($\bar{x}$ = 13.8). The distribution of the participants in this index, shows that 88.9% have an inadequate level; 7.4% have a problematic level and 3.7% have a sufficient level.

**Table 2:** Health literacy indexes.

**Health literacy domain**	**Mean of HL index (SD)**	**Levels of health literacy (%)**			
		**Inadequate**	**Problematic**	**Sufficient**	**Excellent**
General Health Literacy	21.2 (5.0)	85.2	11.1	3.7	0.0
Healthcare	25.3 (6.4)	55.6	37.0	7.4	0.0
Disease prevention	25.0 (4.2)	66.7	25.9	7.4	0.0
Health promotion	13.8 (7.0)	88.9	7.4	3.7	0.0

### Health Literacy Indexes According to age, sex, and Education Level

As shown in Table 3, when we perform a comparative analysis of the means of all indices in terms of age, we find that it is in the higher age classes, namely between 35 and 54 years of age, that the mean score of the indices of general health literacy ($\bar{x}$ = 23. 1), health care-related health literacy ($\bar{x}$ = 26.5), disease prevention ($\bar{x}$ = 26.4) and health promotion ($\bar{x}$ = 17.0) is highest. On the other hand, in relation to sex, we observe that men show higher mean values for general health literacy ($\bar{x}$ = 26.5), health care literacy ($\bar{x}$ = 31.3), health literacy related to disease prevention ($\bar{x}$ = 27.5) and health promotion ($\bar{x}$ = 12.4) than women. In turn, with regard to educational level, the data show that migrants with secondary education have the highest mean values in the indices of general health literacy ($\bar{x}$ = 23.4), health care-related health literacy ($\bar{x}$ = 28.1), health literacy related to disease prevention ($\bar{x}$ = 25.3), and health literacy related to health promotion ($\bar{x}$ = 16.6).

**Table 3:** Health literacy indexes according to age, sex, and education level.

		**Health literacy domain**			
		**General health literacy (GEN_HL)**	**Healthcare (HC_HL)**	**Disease prevention (DP_HL)**	**Health promotion (HP_HL)**
		**Mean**	**Mean**	**Mean**	**Mean**
Age (years)	18–24	19.0	22.9	22.9	11.1
	25–34	20.5	24.9	24.5	12.3
	35–54	23.1	26.5	26.4	17.0
Sex	Male	26.5	31.3	27.5	21.3
	Female	20.3	24.2	24.5	12.4
Education level	Up to the second cycle of primary education	19.4	23.3	24.8	10.8
	Up to the third cycle of primary education	21.6	26.7	21.8	16.3
	High school	23.4	28.1	25.3	16.6
	Higher education	20.6	23.1	26.6	12.9

## Discussion

The people in a migration situation, suffers from a higher vulnerability that is a result of the migration process, and that vulnerability it is increased by the low levels of health literacy. The evaluation of the health literacy indexes in our sample confirms this of which the respondents are examples. The results that we have achieved, confirm that, the level is inadequate in 85.2% participants of our study. In fact, the data from this study are in line with studies carried out in recent years which point to reduced levels of health literacy in the migrant population (23)., however, it is important to mention that, so far, there are few studies in Portugal on health literacy and migrant populations, and knowledge in this area is still limited and needs further studies (23). Given the reduced levels of health literacy of the migrant population, adverse events such as less optimized health behaviors, worse health status and limited access to health care can occur, leading to a situation of vulnerability, and health inequalities (24, 25).

Regarding health care, the results demonstrate that migrants have difficulties either in obtaining health information or in accessing health services, which translates into an inadequate level of health literacy in this index (56%). In fact, many adult migrants do not have access to appropriate health information, and often face difficulties in managing health issues. They are confronted to health staff with inadequate cultural skills and have difficulties managing and understanding the highly complex health systems (26). Additionally, access to health care can be hampered by different barriers, such as the inability to provide guidance in the health system, previous experiences in the health system, existing expectations regarding care, language and cultural barriers, financial resources and beliefs associated with health (27).

On the other hand, the study data demonstrate the existence of inadequate levels, regarding to literacy related to disease prevention and health promotion. In fact, a recent study developed in Europe in the context of a systematic review of the literature on the health literacy of the migrant population in Europe, demonstrated that the reduced level of health literacy among migrants affects the self-management of chronic illness; it provides low levels of adherence to therapeutic interventions resulting from language barriers in understanding the verbal and written information produced by health professionals (teaching, reading information leaflets and drug labels); and increases the difficulties in accessing health services (24).

In this line of ideas, the World Health Organization recognizes as barriers to accessing health care: nationality and legal status in view of remaining in the country, linguistic and cultural differences, administrative barriers, the inability of the health system to adjust in the different countries and the lack of information about health services (28).

In addition, the data show that the highest mean scores on the General Health Literacy, Health Literacy and Health Care, Health Literacy and Disease Prevention, and Health Literacy and Health Promotion indices are presented by male migrants and by migrants with secondary level education. Indeed, it has been identified in some studies that there are differences between men and women in the level of health literacy, with men tending to have higher levels of health literacy than women (29). In fact, it is important to consider future studies regarding the differences between men's and women's health literacy levels. Indeed, the identification of the role of migrant women as family leaders in the health and disease process is essential and should be valued, as women may be the essential link in the dissemination of health information and modification of health management related habits in their families and communities (29, 30). On the other hand, women are subject, throughout their life cycle, to health transition processes that make them more vulnerable, such as issues concerning reproductive and maternal health, for example, limitations in access to contraception, cultural aspects that influence the management of sexual and reproductive health, the barriers in certain regions of the globe to a safe childbirth and pospartum (30), therefore improved knowledge is needed on how these experiences influence or impact Health Literacy in women.

In our study the data obtained show that migrants with a secondary education level have better Health Literacy than migrants with higher education, however, it is important to note that such an observation may be related to the small sample size, so its interpretation should be careful.

Indeed, regarding educational level, some studies point out its significant impact on the level of Health Literacy in Migrant Populations (25, 31), and higher educational levels may be more associated with higher levels of Health Literacy, and therefore in the way people manage their health (31). On the other hand, it is important for future research to reflect on aspects regarding the methodology and organization of the education system in the migrants' countries of origin, as well as the classification system inherent to the educational level, and how these factors impact the Health Literacy levels of migrant populations (25, 31). Additionally, the mentioned aspects, together with the lack of a culturally congruent care model, which concerns an understanding of the social importance and cultural influence on people's health beliefs and behaviors, based on an awareness by the healthcare professionals, influences the existence of equity in access to health care of migrant populations (25, 32).

Having evidence available on the result of the intervention of nurses in the community, combined with the evaluation of the levels of health literacy of the target populations, will allow interventions to be personalized, which will allow us to provide more efficient care to each community, with higher quality, and based on a model of intercultural care that enhances equity in health and the defense of human rights of migrant populations (32).

The World Health Organization also recognized that the Health Literacy is a social determinant of health, a critical one (33). The increasing abilities obtained by higher levels of health literacy, can be achieved by supporting the migrant communities to better understand the health environment that they are inserted. The community specialized nurse can be the facilitating agent of the change.

It is essential to foster and strengthen research on health literacy in the field of nursing, as nurses are key agents in health promotion and health education (34). Community participation is a fundamental strategy, motivated by the empowerment and counseling of the migrant population, to motivate a change in behavior and in paradigm, from the passive group to the active group responsible for decisions that affect their health status.

## Conclusions

This study with an exploratory approach is innovative and highlights the assessment of the level of health literacy in migrants attending primary health care in Portugal. The study showed that many participants have inadequate levels of health literacy in general, and inadequate levels of health literacy related to health care, disease prevention and health promotion. On the other hand, it highlights the importance of promoting equity in access to health information, contributing to the reduction of inequity in access to health by migrant populations.

The present study has some limitations inherent to the small sample size, so that the reading of the results obtained will have to be careful contextualized and not generalized for the migrant population in general. On the other hand, the limited time for data collection contributed to limit the recruitment of participants. More studies need to be conducted to better understand both the health literacy levels of migrant populations, and the factors associated with different levels of literacy as well, and their impact on health equity.

However, our study, although limited by the small number of participants, shows that there are some differences in the mean scores of the health literacy indices under analysis, for example, regarding age, sex and educational level. It is therefore urgent to identify the most vulnerable groups among migrant populations in terms of health literacy, namely women and those with a lower level of education, in order to design and implement actions that correspond to the different needs identified.

Based on the knowledge of the health literacy level of migrant populations, the implementation of interventions culturally adapted to the context and knowledge/experience of migrant populations, will strengthen health equity for migrant populations and is essential to reduce health inequalities. New studies on Health Literacy of migrant populations are needed to know how this populations access, understand, and uses health information to be able to increase the self-manage of their health. In this context, the present study has a higher relevance for the clinical practice of nurses regarding the strategies mobilized to increase health literacy in migrant populations.

## Data Availability Statement

The raw data supporting the conclusions of this article will be made available by the authors, without undue reservation.

## Ethics Statement

The studies involving human participants were reviewed and approved by Ethics Committee of the Regional Health Administration of Lisbon and Vale do Tejo (Process 052/CES/INV/2019). The patients/participants provided their written informed consent to participate in this study.

## Author Contributions

PM and AC conception and design of the study and performed and interpreted the statistical analysis. PM responsible for data collection. AM drafted the manuscript and revised it. All authors read and approved the final manuscript.

## Funding

The research was funded by the CIDNUR.

## Conflict of Interest

The remaining authors declare that the research was conducted in the absence of any commercial or financial relationships that could be construed as a potential conflict of interest. The reviewer KS declared a shared affiliation with one of the authors AC to the handling editor at time of review.

## Publisher's Note

All claims expressed in this article are solely those of the authors and do not necessarily represent those of their affiliated organizations, or those of the publisher, the editors and the reviewers. Any product that may be evaluated in this article, or claim that may be made by its manufacturer, is not guaranteed or endorsed by the publisher.
