# Supplementary material for: Health Literacy and Migrant Communities in Primary Health Care
Source: Front Public Health. 2022 Jan 24;9:798222. doi: 10.3389/fpubh.2021.798222 (PMC8818741; doi:10.3389/fpubh.2021.798222)
Supplement: Supplementary file 1 [file Table_1.DOCX]

Supplementary Material

# Supplementary Tables

| Table 2 \| Percentage Distributions of all HLS-EU-Q health Literacy Items for total (N=27) | | | | | | | | |
| --- | --- | --- | --- | --- | --- | --- | --- | --- |
| Item | **Relation to**  **HLS-EU matrix** | ***On a scale from very easy to very difficult, how easy would you say it is to:* …** | **1. Very difficult**  **(%)** | **2.Difficult (%)** | **Very Difficult and Difficult (%)** | **3.Easy**  **(%)** | **4. Very easy**  **(%)** | **Easy and very easy**  **(%)** |
| 1 | Healthcare/Access Information | *find information about symptoms of illnesses that concern you?* | 3.7 | 55.6 | **59.31*** | 37.0 | 3.7 | 40.7 |
| 2 | Healthcare/Access Information | *find information on treatments of illnesses that concern you?* | 3.7 | 59.3 | **63.0*** | 37.0 | 0.0 | 37.0 |
| 3 | Healthcare/Access Information | *find out what to do in case of a medical emergency?* | 14.8 | 37.0 | **51.9*** | 33.3 | 14.8 | 48.1 |
| 4 | Healthcare/Access Information | *find out where to get professional help when you are ill?* | 0.0 | 11.1 | 11.1 | 85.2 | 3.7 | **88.9**** |
| 5 | Healthcare/  Understand  information | *understand what your doctor says to you?* | 0.0 | 44.4 | 44.4 | 51.9 | 3.7 | **55.6**** |
| 6 | Healthcare/  Understand  information | *understand the leaflets that come with your medicine?* | 11.1 | 74.1 | **85.2*** | 14.8 | 0.0 | 14.8 |
| 7 | Healthcare/  Understand  information | *understand what to do in a medical emergency?* | 11.1 | 48.1 | **59.3*** | 29.6 | 11.1 | 40.7 |
| 8 | Healthcare/  Understand  information | *understand your doctor’s or pharmacist’s instruction on how to take a prescribed medicine?* | 0.0 | 14.8 | 14.8 | 77.8 | 7.4 | **85.2**** |
| 9 | Healthcare/  Appraise  information | *judge how information from your doctor applies to you?* | 3.7 | 40.7 | 44.4 | 55.6 | 0.0 | **55.6**** |
| 10 | Healthcare/  Appraise  information | *judge the advantages and disadvantages of different treatment options?* | 22.2 | 66.7 | **88.9*** | 11.1 | 0.0 | 11.1 |
| 11 | Healthcare/  Appraise  information | *judge when you may need to get a second opinion from another doctor?* | 40.7 | 55.6 | **96.3*** | 3.7 | 0.0 | 3.7 |
| 12 | Healthcare/  Appraise  information | *judge if the information about illness in the media is reliable?* | 0.0 | 33.3 | 33.3 | 59.3 | 7.4 | **66.7**** |
| 13 | Healthcare/  Apply  information | *use information the doctor gives you to make decisions about your illness?* | 3.7 | 25.9 | 29.6 | 66.7 | 3.7 | **70.4**** |
| 14 | Healthcare/  Apply  information | *follow the instructions on medication?* | 0.0 | 7.4 | 7.4 | 85.2 | 7.4 | **92.6**** |
| 15 | Healthcare/  Apply  information | *call an ambulance in an emergency?* | 22.2 | 22.2 | 44.4 | 33.3 | 22.2 | **55.6**** |
| 16 | Healthcare/  Apply  information | *follow instructions from your doctor or pharmacist?* | 0.0 | 7.4 | 7.4 | 85.2 | 7.4 | **92.6**** |
| 17 | Disease  prevention/  Access  information | *find information about how to manage unhealthy behaviour such as smoking. low physical activity and drinking too much?* | 7.4 | 11.1 | 18.5 | 63.0 | 18.5 | **81.5**** |
| 18 | Disease  prevention/  Access  information | *find information on how to manage mental health problems like stress or depression?* | 88.9 | 7.4 | **96.3*** | 3.7 | 0.0 | 3.7 |
| 19 | Disease  prevention/  Access  information | *find information about vaccinations and health screenings that you should have?* | 44.4 | 44.4 | **88.9*** | 11.1 | 0.0 | 11.1 |
| 20 | Disease  prevention/  Access  information | *find information on how to prevent or manage conditions like being overweight. high blood pressure or high cholesterol?* | 7.4 | 3.7 | 11.1 | 77.8 | 11.1 | **88.9**** |
| 21 | Disease  prevention/  Understand  information | *understand health warnings about behaviour such as smoking. low physical activity and drinking too much?* | 0.0 | 7.4 | 7.4 | 81.5 | 11.1 | **92.6**** |
| 22 | Disease  prevention/  Understand  information | *understand why you need vaccinations?* | 0.0 | 18.5 | 18.5 | 77.8 | 3.7 | **81.5**** |
| 23 | Disease  prevention/  Understand  information | *understand why you need health screenings?* | 0.0 | 37.0 | 37.0 | 59.3 | 3.7 | **63.0**** |
| 24 | Disease  prevention/  Appraise  information | *judge how reliable health warnings are. such as smoking. low physical activity and drinking too much?* | 0.0 | 11.1 | 11.1 | 77.8 | 11.1 | **88.9**** |
| 25 | Disease  prevention/  Appraise  information | *judge when you need to go to a doctor for a check-up?* | 18.5 | 63.0 | **81.5*** | 18.5 | 0.0 | 18.5 |
| 26 | Disease  prevention/  Appraise  information | *judge which vaccinations you may need?* | 22.2 | 70.4 | **92.6*** | 7.4 | 0.0 | 7.4 |
| 27 | Disease  prevention/  Appraise  information | *judge which health screenings you should have?* | 25.9 | 63.0 | **88.9*** | 11.1 | 0.0 | 11.1 |
| 28 | Disease  prevention/  Appraise  information | *judge if the information on health risks in the media is reliable?* | 0.0 | 18.5 | 18.5 | 81.5 | 0.0 | **81.5**** |
| 29 | Disease  prevention/  Apply  information | *decide if you should have a flu vaccination?* | 11.1 | 29.6 | 40.7 | 55.6 | 3.7 | **59.3**** |
| 30 | Disease  prevention/  Apply  information | *decide how you can protect yourself from illness based on advice from family and friends?* | 0.0 | 7.4 | 7.4 | 66.7 | 25.9 | **92.6**** |
| 31 | Disease  prevention/  Apply  information | *decide how you can protect yourself from illness based on information in the media?* | 0.0 | 11.1 | 11.1 | 70.4 | 18.5 | **88.9**** |
| 32 | Health  promotion/  Access  information | *find information on healthy activities such as exercise. healthy food and nutrition?* | 0.0 | 0.0 | 0.0 | 77.8 | 22.2 | **100.0**** |
| 33 | Health  promotion/  Access  information | *find out about activities that are good for your mental well-being?* | 88.9 | 3.7 | **92.6*** | 3.7 | 3.7 | 7.4 |
| 34 | Health  promotion/  Access  information | *find information on how your neighbourhood could be*  *more health-friendly?* | 88.9 | 7.4 | **96.3*** | 3.7 | 0.0 | 3.7 |
| 35 | Health  promotion/  Access  information | *find out about political changes that may affect health?* | 96.3 | 0.0 | **96.3*** | 3.7 | 0.0 | 3.7 |
| 36 | Health  promotion/  Access  information | *find out about efforts to promote your health at work?* | 88.9 | 3.7 | **92.6*** | 7.4 | 0.0 | 7.4 |
| 37 | Health  promotion/  Understand  information | *understand advice on health from family members or friends?* | 0.0 | 3.7 | 3.7 | 37.0 | 59.3 | **96.3**** |
| 38 | Health  promotion/  Understand  information | *understand information on food packaging?* | 0.0 | 40.7 | 40.7 | 59.3 | 0.0 | **59.3**** |
| 39 | Health  promotion/  Understand  information | *understand information in the media on how to get healthier?* | 0.0 | 0.0 | 0.0 | 100.0 | 0.0 | **100.0**** |
| 40 | Health  promotion/  Understand  information | *understand information on how to keep your mind healthy?* | 85.2 | 7.4 | **92.6*** | 7.4 | 0.0 | 7.4 |
| 41 | Health  promotion/  Appraise  information | *judge where your life affects your health and wellbeing?* | 77.8 | 11.1 | **88.9*** | 11.1 | 0.0 | 11.1 |
| 42 | Health  promotion/  Appraise  information | *judge how your housing conditions help you to stay healthy?* | 51.9 | 14.8 | **66.7*** | 33.3 | 0.0 | 33.3 |
| 43 | Health  promotion/  Appraise  information | *judge which everyday behaviour is related to your health?* | 40.7 | 22.2 | **63.0*** | 37.0 | 0.0 | 37.0 |
| 44 | Health  promotion/  Apply  information | *make decisions to improve your health?* | 37.0 | 25.9 | **63.0*** | 37.0 | 0.0 | 37.0 |
| 45 | Health  promotion/  Apply  information | *join a sports club or exercise class if you want to?* | 81.5 | 0.0 | **81.5*** | 14.8 | 3.7 | 18.5 |
| 46 | Health  promotion/  Apply  information | *influence your living conditions that affect your health and wellbeing?* | 85.2 | 7.4 | **92.6*** | 3.7 | 3.7 | 7.4 |
| 47 | Health  promotion/  Apply  information | *take part in activities that improve health and well-being in your community?* | 85.2 | 7.4 | **92.6*** | 7.4 | 0.0 | 7.4 |

** Items with representation above 50 in the "very difficult" and "difficult" categories.*

***Items with representation above 50 in the "easy" and "very easy" categories.*
